# State Care of Epileptics

**Published:** 1896-01

**Authors:** F. S. White

**Affiliations:** Late Superintendent State Lunatic Asylum at Austin


### State Care of Epileptics.

LETTER FROM DR. F. S. WHITE, LATE SUPERINTENDENT STATE LUNATIC ASYLUM AT AUSTIN.

*Editors Texas Medical Journal:*

The agitation in the JOURNAL in regard to State care and control of epileptics, is a very timely one. It is an indisputable fact that a person subject to epilepsy is a very undesirable and dangerous member of any community. They are, as a rule, incapable of support, and, as a result, are thrown upon the charity of relatives or friends until they become uncontrollable, when the county authorities take charge of them, and either lock them in jails, send them to their poor farms, or, in very exceptional cases, succeed in having them admitted into one of the insane asylums, where they are the subject of more concern and trouble than any other class of our defective population. The asylums are not the proper places for them, at least not in their present overcrowded condition. Confirmed epileptics are considered as incurables. I do not believe that in my eleven years experience in the asylums of Texas, that I have ever known of a case that was cured, except where the attacks were due to some traumatism, or some reflex trouble;—these cases, however, were exceedingly rare. I have great faith in the future of medicine, and believe that the time will come when this condition will be cured, that is, in recent cases, where no great structural changes have taken place in the nerve centers. There is no doubt but what the attacks are due to an undue accumulation, and, as a result, to an undue and too rapid discharge of nerve force. If some means can be discovered to prevent this accumulation of nervous energy, or, in other words, to secure its regular and equal dissemination throughout the system, the epileptic explosions can not take place. In the meantime, however, while awaiting this Utopian discovery, which will only come by the slow march of scientific research, we must look to the present, and if possible alleviate the afflictions of our failing fellowman, and continue to dose them with bromides and other worthless nerve sedatives. I am strongly of the opinion that the bromides are a positive injury to epileptics. It certainly has nothing curative to commend it. To be sure, these remedies will lessen the number of attacks, but this end is attained at the expense of the patient, whose nervous sensibilities are simply for the time obtunded, and the nerve centers rendered partially inactive.

Enough of this. I did not sit down expecting to say anything about treatment. I believe that the State should take care of these unfortunate creatures, but should do so in connection with her insane population. I advocate this plan only on account of its economy and feasibility. As you know, I have for some time contended, and urged it upon the lawmakers, that the State should assume the care and control of all her insane; that in order to do this in the best and most economical manner a large tract of fertile land should be secured an asylum on the cottage plan erected thereon, where all the patients who are able could be employed in aiding the State in their maintenance. I still advocate this plan, and would have a department of this organization exclusively for epileptics, where they would be properly classified and employed to the best advantage. The whole institution to be under the same management. This would be an ideal colony, and it is what Texas must do, sooner or later, if she proposes to take care of these people on the best and most economical plan.

This plan is no longer an experiment, but is being adopted in a large number of the States. If one will look over the appropriation bill as passed by the last legislature, they will find that the estimated cost of maintaining the three asylums for the years '96 and '97 is $625,660. In addition to this annual expenditure, the State has, at a very low estimate, at least one million dollars invested in these three asylums. And they accommodate something less than 1800 patients. There were in the asylums on October 31, 1894, 1723. The appropriation above referred to is for maintenance of the asylums, including all necessary repairs, etc. A good round sum, but none too large for what has to be done with it. I mention this in order to emphasize the importance of the State adopting the latest and most approved methods of taking care of her insane. These plans are also the most economical.

It costs the people more to care for those cases outside of the asylums now, than it would were the State to assume their care and support. It appears to me that the best interests of the people who pay the taxes would be subserved by these cases being under State control. Their relatives and friends could then devote the time now taken up in attention to then, in useful and profitable employment, and the commonwealth be relieved of a very troublesome and dangerous element, and it would most certainly be best for the afflicted. If the deficit in the treasury disappears, and this subject is properly presented to the next legislature, I believe they will take some action in the matter. I have never yet heard a taxpayer complain of paying his part to support all the eleemosynary institutions. As a rule, the citizen believes that Texas should provide ample and comfortable accommodations for all her insane and epileptics.

I had no idea of saying as much as I have on this subject, but as it is written, will let it go, and if you think worth while, you can publish it; in fact, I believe it might do some good to publish it.

